# The Importance of Acidification in Atopic Eczema: An Underexplored Avenue for Treatment

**DOI:** 10.3390/jcm4050970

**Published:** 2015-05-18

**Authors:** David J. Panther, Sharon E. Jacob

**Affiliations:** Department of Dermatology, Loma Linda University, 11370 Anderson St. Ste. 2400, Loma Linda, CA 92354, USA; E-Mail: dpanther@llu.edu

**Keywords:** atopic dermatitis, skin pH, acid mantle, barrier

## Abstract

Atopic dermatitis is a form of dermatitis commonly seen in children and adults. Its pathophysiology is complex and is centered on the barrier function of the epidermis. An important aspect of the skin’s barrier is pH, which in turn affects a number of parameters such as the skin flora, protease function, and mediators of inflammation and pruritus. Normal pH for non-neonatal skin is acidic and ranges from 4 to 6. Skin pH in atopic dermatitis patients is often increased into the neutral to basic range, and the resulting cascade of changes contributes to the phenotype of atopic dermatitis. Therefore, the maintenance of normal skin pH remains an important topic in understanding and treating atopic dermatitis. This article will review skin pH and its impact on normal barrier function, pathological pH changes in atopic dermatitis, and the therapeutic considerations related to restoring and maintaining pH balance.

## 1. Introduction

Skin pH is being increasingly recognized as a vital component for many of the normal functions of human skin. Although the body’s internal pH tends to be neutral to slightly basic, the normal adult stratum corneum is decidedly acidic with values reported in the 4–6 range [1,2]. This acidification is necessary for antibacterial activity, barrier function, and maturation and structural integrity of the stratum corneum [3]. Dysregulation of the acidity plays a role in a number of diseases, such as candidiasis [4,5], irritant contact dermatitis [6,7], and acne vulgaris [8,9]. One disease in which acidity plays a significantly impactful role is atopic dermatitis [10,11]. This article reviews available literature on how epidermal acidification is achieved, its role in the disease state of atopic dermatitis, and therapeutic options specifically addressing pH aberrancy.

## 2. Biology of Epidermal pH

Acidification of the stratum corneum is achieved and maintained by a number of functional pathways. Secretory phospholipase A2 is a major determinant of pH, acting on phospholipids to generate free fatty acids [12]. The sodium-hydrogen exchanger 1 (NHE1) protein is another important element, causing protons to be pumped into the extracellular compartment at the level of the stratum granulosum [13]. These two mechanisms are likely critical. Another factor in acidification is the processing of filaggrin amino acids to generate polycarboxylic acids [14]. However, it appears that the loss of filaggrin may be compensated for by the first two mechanisms with respect to acidification alone [15], though other detrimental effects may still occur, including altered lipid structure and increased permeability [16]. Additional acidifying factors may include melanin extrusion, as well as sulfated cholesterol [17], and there may be other mechanisms that remain unknown.

A large number of endogenous and exogenous variables are known to affect skin pH, including age, body part, skin type, sweat, soaps, and other topical products [18]. Newborn skin begins with a neutral pH [1], and the transition to acidity occurs during the first year of life, with larger incremental changes occurring in the first two months [19]. The pH again deviates from its normal adult range as it rises in the elderly [20,21], which is likely due in part to a physiologic ceramide deficiency [22]. Body site plays a role in skin pH as well, with infants experiencing higher pH on extensor surfaces and prominences, such as the cheeks and buttocks [23]. Later in the normal adult skin, the intertriginous zones tend to have the highest pH [24].

With any discussion about the generation of acid pH, one must also consider the maintenance of it. Two concepts are important here: the capacity of intrinsic acidification mechanisms to respond to an aberration, and the buffering capacity of the skin. As discussed above, phospholipase A2 and NHE1 seem capable of being upregulated in response to the loss of filaggrin-induced acidification, and would be expected to be capable of responding to other insults as well. The buffering capacity is largely dependent on free amino acids, which are in part derived from filaggrin byproducts [25]. The skin’s ability to buffer against strongly acidic or alkaline substances is critical for protection against brief environmental insults, though prolonged contact with such substances will easily overwhelm the buffer.

The acidic pH is critical to the overall function of the skin, and a number of specific mechanisms have been elucidated which highlight its importance. The skin’s antimicrobial properties are optimal at acidic pH. *Staphylococcus* and other pathogenic bacteria favor neutral pH and are inhibited in an acidic milieu [26,27,28]. In addition, antimicrobial proteins produced by keratinocytes, such as dermicidin, are more effective in an acidic stratum corneum [29,30]. Desquamation of the stratum corneum is a controlled process critical for ensuring correct thickness, and a key factor is enzymatic degradation of corneodesmosomes by serine proteases, particularly kallikreins 5 and 7 [31,32,33]. These enzymes act on desmoglein 1, and the process is relatively slow at acidic pH [34]. When pH rises to the enzymes’ optima (neutral), the degradation occurs much more briskly, leading to inappropriate desquamation and decreased integrity of the stratum corneum [35,36]. Another function of kallikrein 7 is to activate IL-1 beta [37], which has also been noted to increase at neutral pH, initiating or perpetuating chronic inflammation.

Ceramide production is yet another pH dependent process, with acid sphingomyelinase and beta-glucocerebrosidase functioning best at a pH of 4.5 and 5.6, respectively [38]. Increases in pH have been shown to reduce their enzymatic activity and impair barrier function, which ceramides are critical for maintaining [35].

3. pH in Atopic Dermatitis

A number of interconnected mechanisms come into play in the atopic dermatitis disease state, and pH is certainly one of the critical players. Lesional skin has been shown to have the highest pH on a given patient with atopic dermatitis, followed by perilesional skin being somewhat more acidic, and unaffected skin having the lowest pH [10,11]. Even unaffected skin in atopics has a higher pH than normal controls [39]. Clinical evidence for the importance of pH is evidenced by the sites of predilection, with the less acidic areas having a greater propensity for disease expression. In infancy, the cheeks tend to have a higher pH, corresponding to atopic dermatitis favoring this area [23]. However, intertriginous and flexural skin has the more elevated pH in adult skin [24], again corresponding to the favored sites in this age group.

Although the downstream pathways dependent on pH are relatively well-defined, the upstream causes of the pH increase seen in atopics remain less clear. A number of possibilities have been proposed, both endogenous and exogenous. As mentioned above, filaggrin likely plays a role in acidification and its mutation is widely known to be a significant risk factor for atopic dermatitis. It has not been conclusively shown whether its importance in this disease relates more to its role in acidification *versus* its role in barrier and lipid homeostasis. Changes in one or both of the two more pivotal acidification mechanisms, phospholipase A2 and the NHE1 protein, may also occur but have not been specifically measured in the disease state. Further study is needed to determine if and how these mechanisms are altered. Another potential source of pH aberration is extracellular fluid (ECF). The barrier function of the stratum corneum must not only be thought of as a barrier to external insult, but also as an internal sequestration of a uniquely acidic microenvironment. Compromise of the barrier may lead to undue influence from ECF, even in the absence of overt erosion.

In addition to the body’s intrinsic pH variability, external variables also directly affect development and severity of atopic dermatitis. Given atopic patients’ decreased buffering capacity [25], external influences are magnified in this population. Alkaline soaps are known to induce lesions of atopic dermatitis in susceptible individuals [40,41]. Hypochlorite certainly would be expected to increase pH although it has a secondary effect of being antimicrobial, which improves one of the key negative consequences of elevated pH, bacterial colonization. Hypochlorite has been shown to be beneficial in its overall effect, when used appropriately [42]. At an approximate dilution of 1:1000 for 6% sodium hypochlorite with an original pH of around 12, the pH of the bath can be estimated to be around 9. The precise effect bleach baths have on skin pH has not been quantified.

## 4. Acidic pH as a Therapeutic Goal

Given the importance that physiologic acidic pH plays in the integrity, barrier function, and antimicrobial properties of the skin, it is naturally becoming a therapeutic target. While the literature is replete with reviews and studies on how pH contributes to the overall physiology of the skin, there is a paucity of current evidence on how to clinically approach pH management. The two primary avenues of treatment opportunities for pH management are bathing parameters (soap, temperature, time) and the moisturizer applied to skin on a regular basis. While other factors may contribute, such as occlusion and environmental humidity, their effects on pH are not well defined.

The last review of the pH of common commercial cleansers was done in 2002 [43], and while it is enlightening, it cannot be relied on for informing current recommendations to patients. Hand washing with alkaline soap can increase the pH of the skin by 3 pH units, and that effect persisted for 90 min [38]. The effects on pH are assumed to be primarily responsible for the provocation of atopic dermatitis after using neutral or alkaline soaps [40,41], although the stripping of protective lipids may be another mechanism by which soaps perpetrate their damage. It would be beneficial to have more data available regarding the pH of currently available cleansers. In addition, the utility of considering pH in cleansers would be solidified by well-designed studies comparing those of varying pH, and quantifying both the immediate pH effects and the long-term clinical benefit of sustained use in atopic patients.

Topical emollient and leave-on products represent the other key opportunity for intervention. Colloidal oatmeal has long been known to be beneficial in atopic dermatitis. It has been recognized as acting, in part, through normalizing skin pH to its usual acidic range [47]. It remains a commonly found ingredient in currently available topical preparations for atopic dermatitis. Very little is known about the pH of currently available products, although evidence is mounting for the potential benefits of acidic emollients in treating and even preventing atopic dermatitis. In 2009, it was first shown that maintaining an acidic pH with polyhydroxyl acids can actually prevent the emergence of atopic dermatitis in a murine model of oxazolone-induced disease [44]. Lee *et al.*, recently used a murine model to show the prevention of atopic dermatitis and subsequent atopic march to asthma [45]. They compared an acidified Cetaphil cream (pH 2.8) with the same Cetaphil cream after neutralization (7.4) twice daily while attempting to incite skin lesions of atopic dermatitis with topical oxazolone and airway disease with *Dermatofagoides pteronyssinus* antigen. Significant differences were seen in both skin and airway, favoring the acidification cream. Application of these results to a human subject experiencing multiple and varying insults throughout the day may not be possible, but the results are intriguing nonetheless. Again working in a murine model, Hachem *et al.*, showed a wide variety of positive influences on the stratum corneum by application of polyhydroxyl acids [46]. These effects included improved barrier homeostasis and supranormal stratum corneum integrity and cohesion. It remains to be seen precisely how these data may translate to the understanding and treatment of atopic dermatitis in humans, but the data are mounting that active management of pH is vital to the treatment and possibly primary prevention of this common condition.

## 5. Novel Concepts in Intervention

The benefits of restoring and maintaining the acid pH of the skin have been known for decades, but there is still a great opportunity for advances. One key question that remains to be answered is whether particular acids have specific benefits above and beyond simply correcting pH abnormalities. Lee’s comparison of acidified *versus* neutralized versions of the same moisturizer emphasizes the importance of acidification alone [45], although the citric acid used to lower pH does have its own unique effects such as epidermal thickening [48]. The replenishing of bio-similar or bio-identical acids or non-native acids with known biological activity is an intriguing prospect. Improvement in atopic dermatitis has recently been reported using *cis*-urocanic acid [49], which is known to suppress the induction of immunity in skin [50]. As discussed above, polyhydroxyl acids improve a number of parameters [46], though it is difficult to be certain whether these effects could have been caused by acidification alone, irrespective of the acid used. Specific antibacterial effects have not been well characterized for the acids currently under investigation, but this could be an additional property of some acids that would make them superior in the therapy of atopic dermatitis. Another aspect of treatment, particularly in compromised lesional skin, is the potential for irritation to be caused by a topical therapy. Targeting this effect could represent a way to make irritating but beneficial therapies more tolerable. Strontium has been shown to be an inhibitor of irritation in the skin [51], and one study has shown significant improvement in stinging and burning when strontium was included in one of the tested acid preparations [52]. Unfortunately, as noted above, the pH of currently available topical OTC and prescription products is almost entirely unknown. In addition, the complex chemical formulation of most products will make it difficult to predict irritant potential based on pH alone. More investigation into strontium and other anti-irritant compounds could lead to improved tolerability and compliance with topical therapies.

## 6. Conclusions

The acid mantle of the skin has been known for decades, though its roles in various aspects of skin physiology have only recently been elucidated. Acidity is critical for ensuring appropriate stratum corneum integrity, barrier function, and antimicrobial activity. Maintaining a normal pH through appropriate therapy is well known to benefit disease states such as atopic dermatitis, and acidification may even have preventative effects when instituted prior to onset of the atopic phenotype. There are many opportunities for enhancing the current body of knowledge in this area, including updating the published pH values for available cleansers and emollients, as well as exploring new acids for use in therapy. The consideration of pH normalization will continue to be an increasingly important aspect of a comprehensive treatment plan in atopic dermatitis and other cutaneous diseases.
